# Morphological Effect of Non-targeted Biomolecule-Modified MNPs on Reticuloendothelial System

**DOI:** 10.1186/s11671-015-1075-0

**Published:** 2015-09-17

**Authors:** Xiao Li, Yan Hu, Jie Xiao, Dengfeng Cheng, Yan Xiu, Hongcheng Shi

**Affiliations:** Department of Nuclear Medicine, Zhongshan Hospital, Fudan University, Shanghai, China; Shanghai Institute of Medical Imaging, Shanghai, China

**Keywords:** Magnetic nanoparticles, Morphological effects, Reticuloendothelial system, Cellular uptake, Biodistribution

## Abstract

Magnetic nanoparticles (MNPs) with special morphology were commonly used as biomaterials, while morphological effects of non-targeted biomolecule-modified MNPs on biological behaviors were still unclear. In this research, spherical and rod-like Fe_3_O_4_ in a comparable size were synthesized and then surface-modified by bovine serum albumin (BSA) as a model of non-targeted biomolecule-modified MNPs. Morphological effects were featured by TEM and quantification of in vitro phagocytic uptake, as well as the in vivo quantification of particles in reticuloendothelial system (RES)-related organs of normal Kunming mice. For these non-targeted BSA-modified MNPs, intracellular distributions were the same, but the rod-like MNPs were more likely to be uptake by macrophages; furthermore, the BSA-modified MNPs gathered in RES-related organs soon after intravenous injection, but the rod-like ones were expelled from the lung more quickly and expelled from the spleen more slowly. These preliminary results may be referable if MNPs or other similar biomolecule-modified nanoparticles were used.

## Background

Targeting delivery to diseased focus, as well as decreasing side effect to metabolic organs, is the main aim of nanoparticle-based drug delivery system. As an important item of biomedical materials, magnetic nanoparticles (MNPs) were often used as magnetic and biological targeting delivery system for theragnostic agents [1, 2]. For example, RGD-functionalized MNPs target for integrins of tumorous vascularization [3]; HIV-derived TAT-conjugated MNPs lead to efficient T cell labeling [4]. However, most nanoparticles concentrate in reticuloendothelial system for in vivo applications, decreasing targeting efficiency. On the other side, when being used in hyperthermia [5], accurate understanding of biodistribution is essential to avoid thermal injury to normal tissues.

Morphology heavily influences targeting efficiency, as well as pharmaceutical effects. Research on nanoparticle-based delivery systems with special morphology often aims to extend cycling time, increase targeting efficiency, and decrease damage to normal tissues, so as to improve pharmaceutical effects. However, morphological effects on the uptake of reticuloendothelial system (RES)-related organs were often neglected. Hence, figuring out the morphological effect on biological behaviors is of pragmatic value for biomedical applications.

In this research, bovine serum albumin (BSA)-modified MNPs with similar size but different morphologies were synthesized to feature the corresponding morphological effect on reticuloendothelial system.

## Methods

### Synthesis of BSA-Conjugated MNPs

**Fig. 1:**
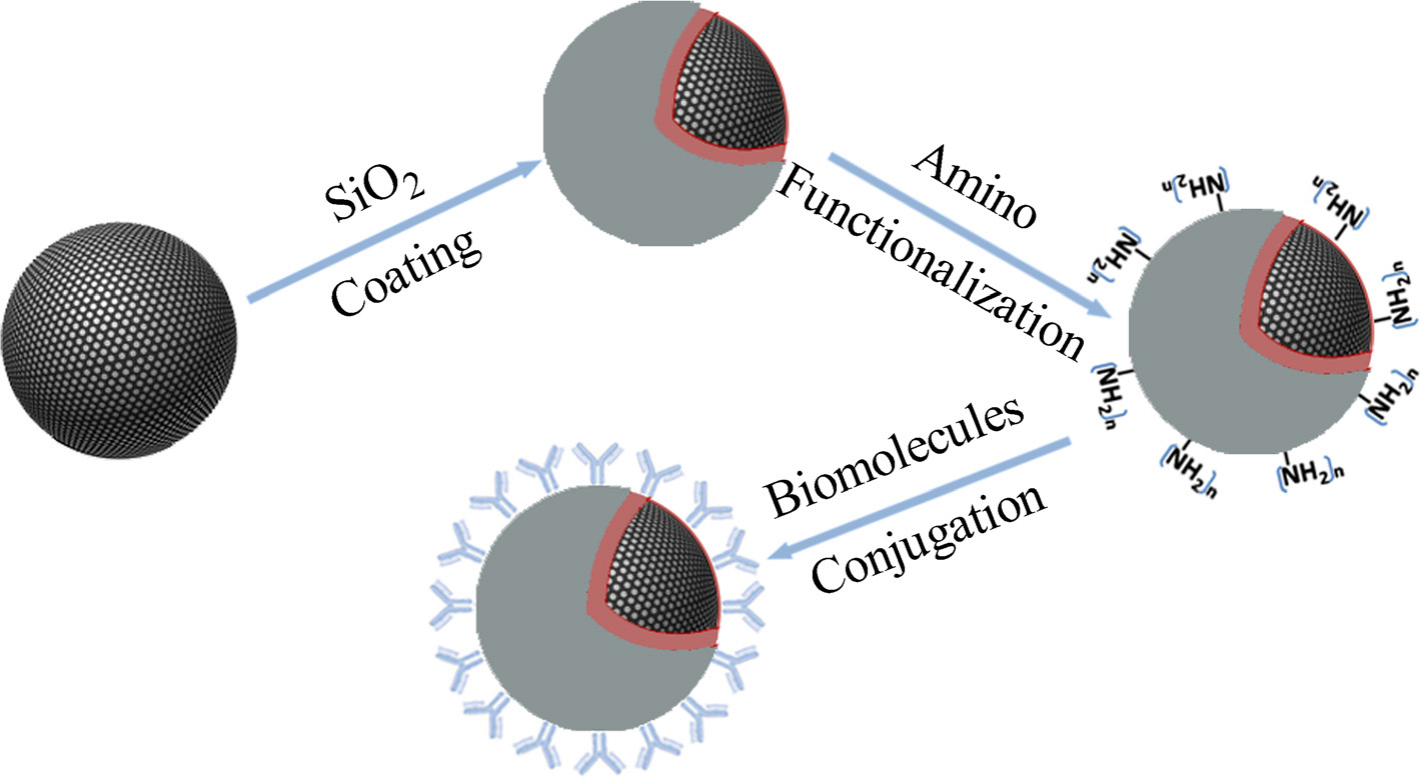
A representative procedure of synthesis of biomolecule-modified MNPs. Specifically, SiO_2_ surface coating, amino functionalization, and biomolecules conjugations

Spherical Fe_3_O_4_ was synthesized via hydrothermal method: 0.81 g FeCl_3_·6H_2_O and 1.47 g KAc were dissolved in 25 mL ethylene glycol and then treated by ultrasound for 1 min. The mixture was sealed in a 50 -mL autoclave and maintained at 200 °C for 24 h. Product was washed by water and alcohol for three times and collected by a magnet. Rod-like Fe_3_O_4_ was synthesized following a reported hydrothermal method from Y. Liu [6]. According to G. Zhang’s method (referred to Fig. 1), Fe_3_O_4_ was completely covered by SiO_2_ via alcoholysis of tetraethyl orthosilicate (TESO) in a mixed solution of methanol and methylbenzene (1:1, *v*/*v*) and then amino-functionalized via alcoholysis of N-(2-aminoethyl)-3-aminopropyltrimethoxysilane (AEAPS) [7]. Finally, BSA was conjugated to MNPs via reaction between amino groups and glutaric dialdehyde-activated amino-modified MNPs at 4 °C for 24 h. For in vivo tracking of MNPs’ distribution, radiotracer-^125^I was labeled to proteins: 3.7 MBq Na^125^I and BSA-modified MNPs (1 mg MNPs contained) reacted for 5 min at 25 °C with the catalysis Iodogen.

X-ray diffraction (XRD) was performed at an operation voltage of 40 kV and a current of 40 mA with Cu Kα radiation (*λ* = 0.154056 nm). TEM (operated at 200 kV) was used to picture the size and morphology of MNPs. FTIR was used to verify the existence of –NH_2_; I-125 labeling rate was measured by radio-TLC with saline as the developing solvent. Stabilities in PBS and 10 % fetal bovine serum (FBS) were measured for 24 h at most.

### Uptake Efficiency of BSA-Conjugated MNPs

Raw 264.7 macrophages were used for in vitro cellular uptake test. The macrophages were cultured in Dulbecco’s Modified Eagle Medium (DMEM) with 10 % FBS at 37 °C in 5 % CO_2_ atm. and seeded at 10^5^ cells/well in a 96-well plate. After 2 h incubation, the cells were exposed to a culture medium containing MNPs at 20 μg/mL for 12 h at most. For cellular uptake test, unfixed nanoparticles were rinsed with 0.01 M PBS, and macrophages were then dissolved by HCl. Quantification of MNPs was determined via absorbance measurement of complex of ferrozine and ferrous ion at 562 nm and then corresponded to the standard curve. For TEM, the macrophages were fixed by 4 % paraformaldehyde, dehydrated by alcohol at gradient concentration, embedded into epoxy resin, and then cut into slices of 75 nm.

### In Vivo Biodistribution

Animal care and all experimental procedures were performed under the approval of the Animal Care Committee of Fudan University. Forty Kunming mice (male, 20 ± 1 g) were selected for in vivo biodistribution research. The BSA-modified MNPs (0.74 MBq) were injected intravenously. The mice were scarified at 20 min, 1 h, 4 h, and 12 h (five mice for each group) after injection. RES-related organs were collected, weighted, and measured for radioactivity. Biodistribution was expressed as percentage of injected dose per gram (% ID/g), and differences between the spherical and rod-like particles were analyzed with paired *t* test.

## Results and Discussion

**Fig. 2:**
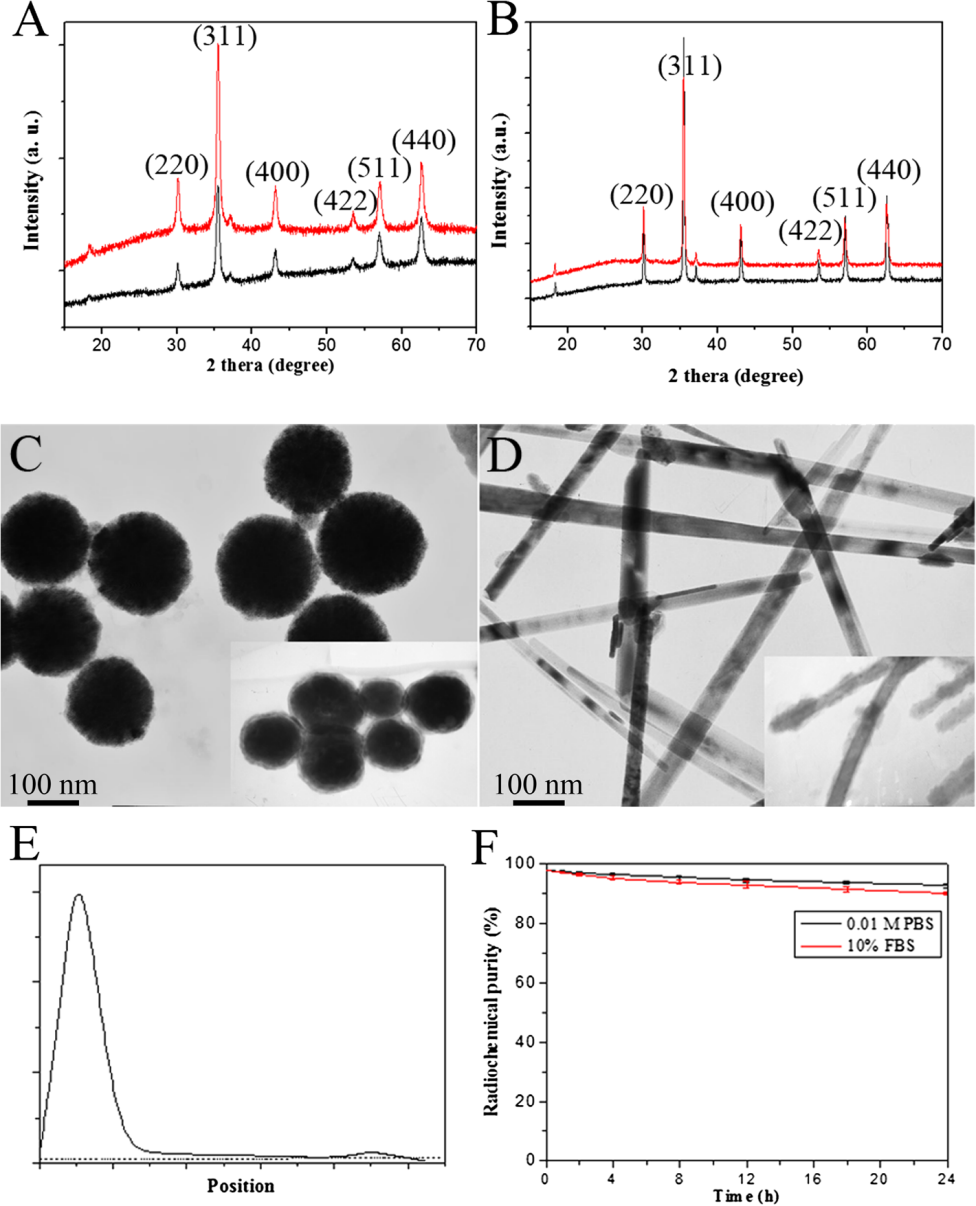
Characteristics of BSA-modified MNPs. XRD spectrum of Fe_3_O_4_ and SiO_2_@Fe_3_O_4_ (the *red lines*) for spherical MNPs (**a**) and rod-like MNPs (**b**). TEM of MNPs for spherical MNPs (**c**) and rod-like MNPs (**d**) with TEM of SiO_2_-coated MNPs on the *lower right corner*. Radio-TLC (**e**) and stability (**f**) of I-125-labeled BSA-modified MNPs

Products from the two hydrothermal processes shared a same XRD spectrum of pure Fe_3_O_4_ (JCPDS No. 19-0629), meanwhile, the relevant stronger peak value of the rod-like Fe_3_O_4_ means an orientated growth along (311) crystal face. SiO_2_ coating is amorphous, reflected by “bread”-shaped scattering peaks at around 25° (Fig. 2a, b). MNPs based on the different hydrothermal processes were totally different on morphology (Fig. 2c, d). The diameter of the spherical MNPs was 150–200 nm, while the length of the rod-like MNPs was 200–400 nm and has a diameter of 20–30 nm. SiO_2_ coating covered the MNPs evenly with a thickness of <10 nm. The characteristic peak existed in 3380 and 3186 cm^−1^ in FTIR spectrum proved the existence of –NH_2_ after alcoholysis of AEAPS. I-125 was labeled to proteins stably with a radiochemical purity of >98 % and kept stable in PBS and 10 % FBS for more than 24 h, guarantying truly reflection of in vivo biodistribution of BSA-conjugated MNPs (Fig. 2e, f).

Nanoparticle-based drug delivery systems are often spherical and with a diameter of 100–200 nm, such as the FDA-approved doxorubicin liposome (Doxil) and liposome-based Abraxane [8]. As a representative morphology, the rod-like MNPs were chosen to reflect the morphological effects on biological behaviors. The as-synthesized particles matched the optimal size for potential in vivo application. BSA was used as the non-targeted model of biomolecules, avoiding the confounding factor of targeting and emphasizing biodistribution resulted from morphological effects. I-125 was directly labeled to tyrosine of proteins; low-energy γ-ray can be easily detected by a radio detector but does not lead to self-radiolysis [9].

### In Vitro Cellular Uptake

**Fig. 3:**
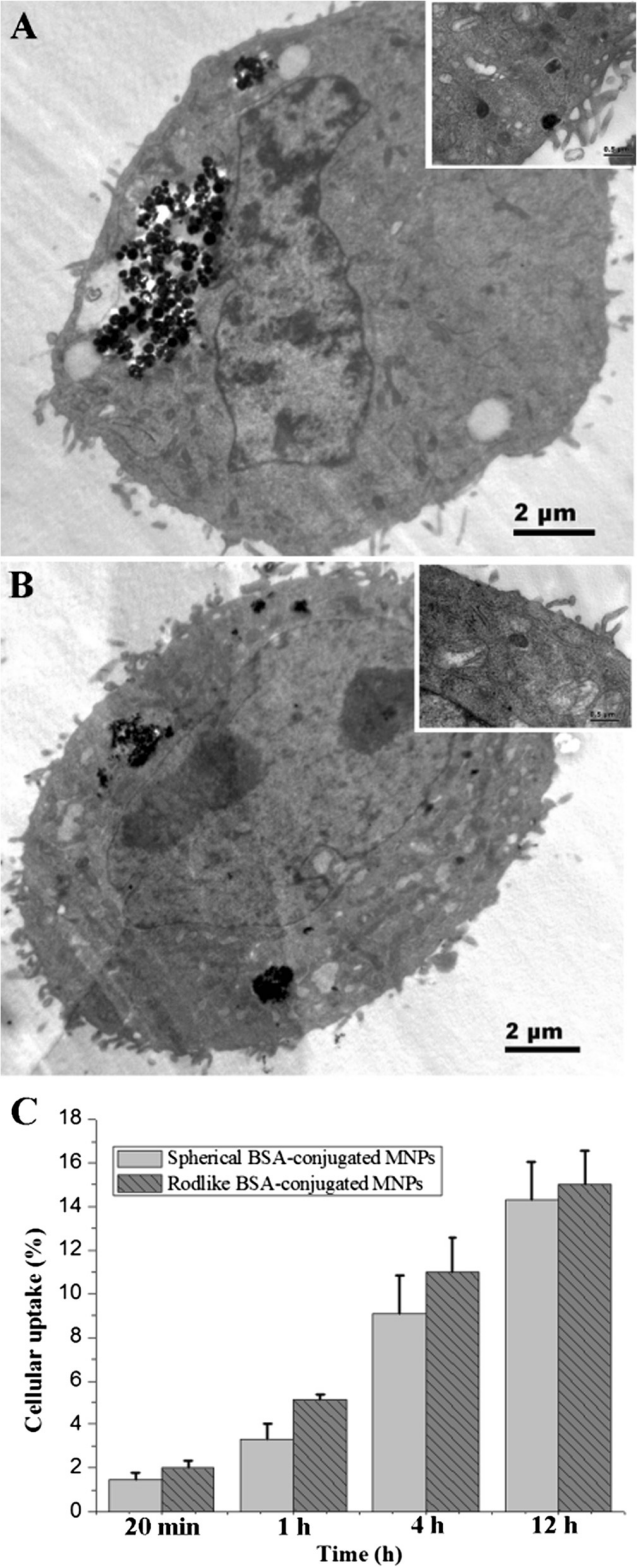
TEM and quantification of cellular uptake. TEM of cellular uptake of spherical (**a**) and rod-like (**b**) BSA-modified MNPs. Quantification of cellular uptake (**c**)

These non-targeted particles were uptake via endocytosis mainly. The MNPs were observed in the cytoplasm in clusters, and nearly no MNPs entered the nucleus (Fig. 3a, b). There was a significant difference (*P* < 0.05) on uptake efficiency during the early period of uptake, but the difference tended to decline with time and changed to a non-significant difference (*P* = 0.378) after 12-h incubation (Fig. 3c).

Samir Mitragotri pointed out that morphology has a direct influence on endocytosis, promoting, inhibiting, and cellular uptake [10]. This minor in vitro difference observed above between the spherical and rod-like MNPs may be amplified for viviperception.

### In Vivo Biodistribution

**Fig. 4:**
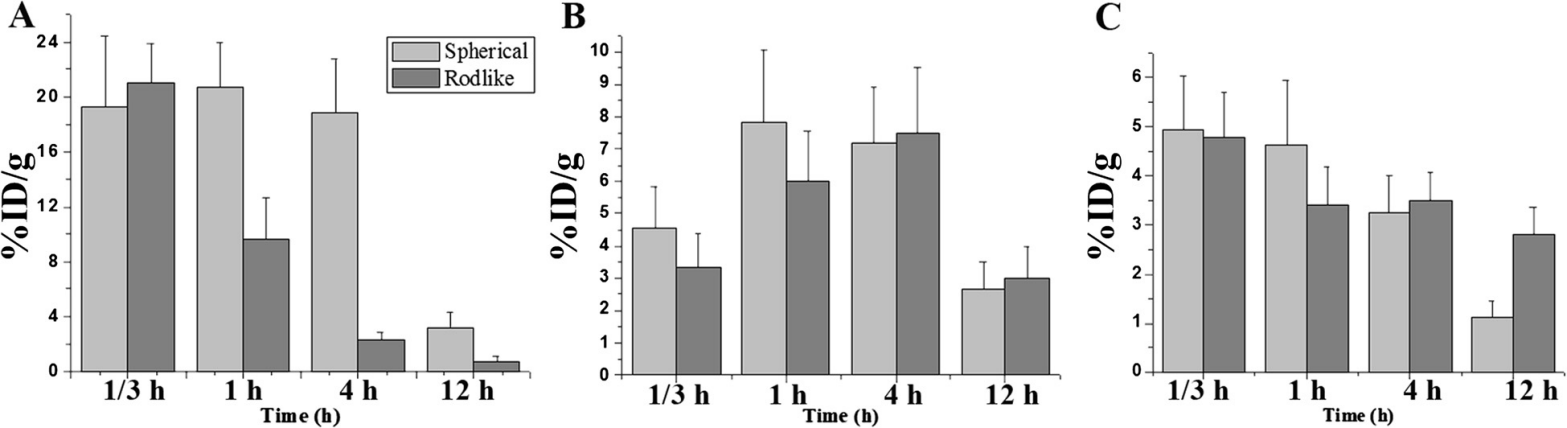
Uptake efficiency and expelling rate of BSA-conjugated MNPs by RES-related organs. Specifically, the lung (**a**), liver (**b**), and spleen (**c**)

For in vivo biodistribution, MNPs gathered in RES-related organs soon after injection (19.2 % ID/g for the spherical ones, 20.9 % ID/g for the rod-like ones). Liver uptake got the peak value (7.8 % ID/g for the spherical ones and 7.6 % ID/g for the rod-like ones) at around 1–4 h after injection, and the particles were expelled at a similar rate. Due to the morphological effects, the rod-like BSA-modified MNPs were expelled from the lung more quickly, meanwhile, the rod-like particles were expelled from the spleen more slowly (Fig. 4).

Most non-targeted biomolecule-modified nanoparticles accumulated in the reticuleoendothelial system because of endocytic effect of macrophages. Besides RES uptake, asialoglycoprotein receptor on hepatic cells which recognize galactose residues and acetyl galactosamine residues on biomolecules [11] partially contribute to uptake. Morphological effects were mainly reflected by metabolic differences on the expelling rate, which were resulted from the macrophages percentage composition and difference on microstructures of tissues. Taking the lung as an example, extraneous nanoparticles were easily held by pulmonary alveolus, but particles with special morphology tends to be released more quickly than the spherical ones. Hence, morphological effects, as well as targeting efficiency, should be overall considered when designing a biological targeting nanoparticle-based drug delivery system.

## Conclusions

The BSA-modified MNPs with typical morphologies were synthesized. Both the spherical and rod-like biomolecule-conjugated MNPs gathered in RES-related organs soon, but the rod-like MNPs tend to be expelled from the lung more quickly but expelled from the spleen more slowly.
